# Metachronic distal rectovaginal septum metastasis with prior laparoscopic anterior resection for proximal rectal carcinoma

**DOI:** 10.1093/jscr/rjad303

**Published:** 2023-05-20

**Authors:** Thaís T T Tweed, Kayleigh A M van Dam, Meindert N Sosef, Henricus J Belgers

**Affiliations:** Department of Surgery, Division of Gastro-Intestinal Surgery, Zuyderland Medical Center, Sittard-Geleen, Heerlen, The Netherlands; Department of Surgery, Maastricht University Medical Centre, Maastricht, The Netherlands; Department of Surgery, Division of Gastro-Intestinal Surgery, Zuyderland Medical Center, Sittard-Geleen, Heerlen, The Netherlands; Department of Surgery, Division of Gastro-Intestinal Surgery, Zuyderland Medical Center, Sittard-Geleen, Heerlen, The Netherlands; Department of Surgery, Division of Gastro-Intestinal Surgery, Zuyderland Medical Center, Sittard-Geleen, Heerlen, The Netherlands

## Abstract

Metastatic disease in the vagina of other origins such as rectal cancer is rare and only very few cases have been reported. A female patient developed an isolated metachronic metastasis located at the lower part of the rectovaginal septum, 8 months after curative resection for proximal rectal cancer. An excision of the tumour was performed with primary closure of the vaginal wall. Histopathological examination confirmed the solid tumour to be metastatic disease from rectal origin with free margins. A year later, the patient received a lobectomy of the left lower lobe, due to distant metastasis of rectal origin 2 years after primary surgery. The patient is currently 4 years postoperatively, alive and shows no sign on recurrent disease. This case illustrates that awareness and early recognition of this rare presentation can lead to adequate treatment plans.

## INTRODUCTION

Cancer located in the vagina is considered to be a rare entity in the scope of all known cancers. Worldwide, vaginal cancer location encompasses ⁓1–3% of all gynaecological malignancies diagnosed in women [1]. In the Netherlands ⁓64 new cases are registered each year of isolated or invasive primary vaginal cancer with a 5-year survival chance of 44-95%, depending on the tumour stage upon diagnosis [2]. The majority of cases of vaginal cancer are due to metastatic disease, most originating from the endometrium, cervix or ovaries [3]. Metastatic disease in the vagina of other origins is rare and only very few cases have been reported in (English) literature. In this report, we present a case of a patient in her early 50s with an isolated metachronic metastasis located at the lower part of the rectovaginal septum, 8 months after curative resection for proximal rectal cancer.

### Case description

We present the case of a female patient who was referred to us by the gastroenterologist. The patient had been previously evaluated for symptoms of rectal blood loss and changes in passing stools. A colonoscopy revealed a rectal tumour at 14 cm from the anal verge. Histopathological investigation confirmed the diagnosis of a well-defined adenocarcinoma. Rectal magnetic resonance imaging (MRI) showed a clinical T2N2 stage tumour with a length of 4.2 cm, located 14 cm from the anorectal junction. The mesorectal fascia was not threatened, with seven suspected mesorectal lymph nodes. There were no signs of extramural vascular invasion or suspected extramesorectal lymph nodes. There was no sign for disseminated disease.

The patient had no relevant previous medical history. She was counselled for neoadjuvant chemoradiation with Xeloda during which her menopause started. The Xeloda was discontinued due to cardiac side-effects. A restaging MRI was performed 8 weeks after ending of the neoadjuvant treatment which showed minimal reduction of tumour size resulting in a clinical T2N2 stage tumour.

A laparoscopic anterior resection with a primary end-to-end anastomosis at 10 cm of the anal verge without a diverting ileostomy was performed. Her postoperative recovery was uneventful and she was discharged on day 3 postoperatively. The histopathological examination revealed a radical resected well differentiated adenocarcinoma with a diameter of 1.7 cm, with maximal depth invasion into perirectal fatty tissue and three out of 12 positive lymph nodes, ypT3N1. Tumour regression grading score of three according to the Mandard tumour regression grade [4].

During the regular oncological follow-up 8 months post-surgery she mentioned newly discovered postmenopausal and postcoital vaginal blood loss, local discomfort and mild urinary incontinence. The carcinoembryonic antigen (CEA) level was 3.2 μg/l, and the abdominal ultrasound showed no signs of liver metastasis. Because of these new symptoms, she was referred to a gynaecologist who diagnosed a tumour at a short distance of the introitus on the posterior wall of the vagina. This tumour was of approximately one centimetre in diameter and had a firm, elastic character. Further gynaecological examination showed no abnormalities.

### Investigations, treatment and follow-up

**Figure 1:**
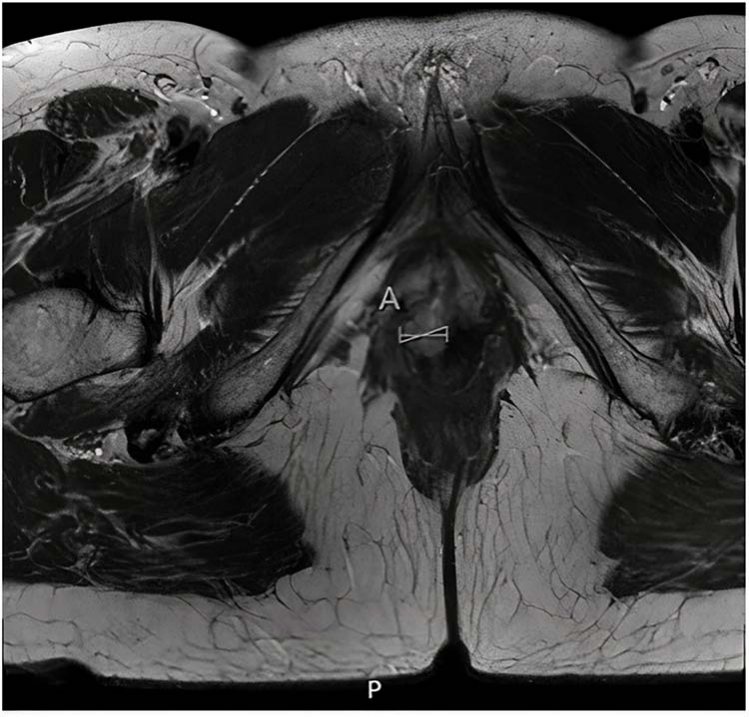
Axial view of diagnostic MRI-rectum with the solid tumour indicated with the linear mark ‘A’.

**Figure 2:**
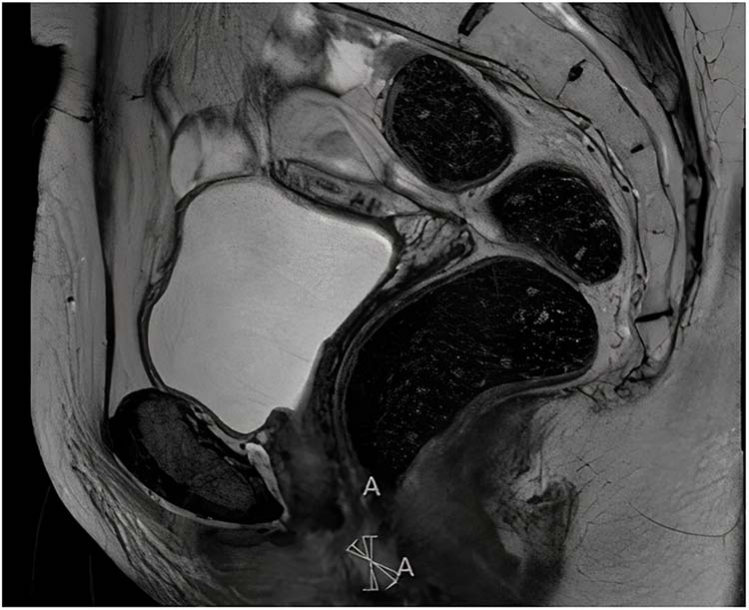
Sagittal view of diagnostic MRI-rectum with the solid tumour indicated with the linear mark ‘A’.

Histopathological examination of the biopsy showed an adenocarcinoma metastasis of rectal origin. A full body positron emission tomography (PET)-scan and MRI were made and confirmed the vaginal lesion of 1.7 × 1.4 × 1.5 cm located in the posterior wall of the vagina just above the puborectal sling (see Figs 1 and 2). From the histopathological and imaging diagnostics we concluded that the patient had a distal rectovaginal septum metastasis of rectal origin.

**Figure 3:**
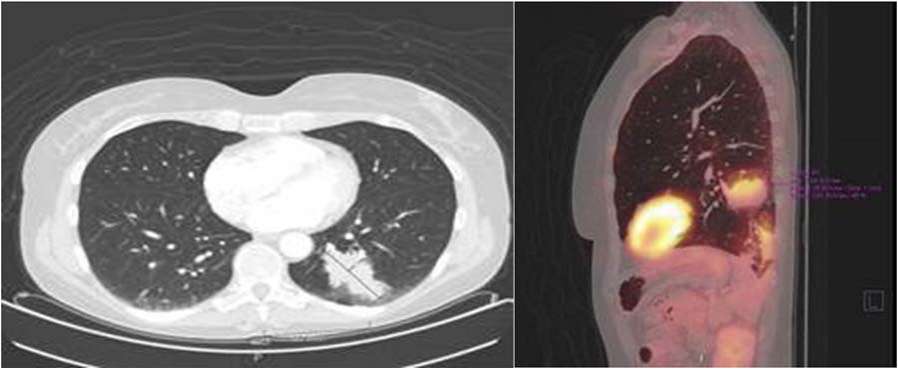
Transverse- and sagittal plane of the PET-scan showing a leasion in the left lower lobe with metabolic activity.

After 1 month of initial diagnosis, an excision of the tumour was performed through open approach with primary closure of the vaginal wall. Histopathological examination confirmed the solid tumour to be metastatic disease from rectal origin with free margins. The newly discovered symptoms related to the rectovaginal septum metastasis had completely disappeared following the excision. Upon further follow-up at 2 years and 6 months; routine lab results showed a significant increase in the CEA levels at 5.2 ug/l (3 months prior at 4.0 ug/l). Given this significant increase; a PET-scan was again performed. The PET-scan revealed a lesion within the left lower lobe of 5.7 cm in a transverse plane; suspected to be distant metastasis (see Fig. 3). Compared to the last PET-scan, this lesion was a new development not previously seen. CT-guided thoracic biopsy confirmed by histopathological examination, that this lesion was indeed a distant metastasis of the previous rectal cancer.

The patient was then electively operated and received a lobectomy of the left lower lobe via the Uniportal Video-Assisted Thoracoscopic Surgery method. This was an uncomplicated procedure and after 2 days, the patient was then discharged. The patient is currently 4 years postoperatively (primary surgery) and in follow-up. The last CT-Thorax/abdomen showed no signs of recurrent malignancy (13.01.2021) and the most recent CEA value was 3.7 ug/l (21.10.2021). The patient is currently alive.

## DISCUSSION

Vaginal tumours are rare, compromising ⁓1% of all tumours worldwide. In this group, secondary tumours compromise ⁓80% of all cases, presenting themselves as distant metastases [5, 6]. Most tumours originate from the gynaecological tract (54%) and about (46%) from extra-gynaecological structures [5]. Very few cases of vaginal tumours secondary to (colo)rectal malignancies have been reported. Several authors have hypothesized over the plausible routes and/or mechanism of metastases, all with little supporting evidence [6–8]. These hypotheses include (i) the iatrogenic route; due to infiltration and migration of cancerous cells during surgery via de pouch of Douglas. (ii) Through retrograde flow in the lymphatic pathways; rectal- and (several) vaginal lymph vessels come together and drain into the sacral and rectal lymph nodes. (iii) Through arterial and venous flow; cancerous cells could migrate from the rectum to the vaginal venous plexus. (iv) Also, through migration via the fallopian tubes; by dissemination of cancerous cells in the intraperitoneal space.

Vaginal tumours could go undetected, as most vaginal tumours are asymptomatic until a later stage. Sadatomo *et al.* summarized the most common forms of presentation and symptoms of patients with vaginal tumours. Vaginal bleeding is by far the most common primary symptom in patients presenting either postmenopausal or postcoital [8]. Alongside this symptom, pelvic pain and the sensation of an intra-cavity mass are also common symptoms [9].

Current diagnostic standards of colorectal cancer do not include additional gynaecological examination, nor imaging for the gynaecological tract in asymptomatic patients. Medical professionals should consider a full gynaecological examination with or without extending medical imaging to the gynaecological tract for female patients who present with colorectal cancer and symptoms of the gynaecological tract. Early diagnosis and timely treatment could lead to an increased survival chance as the 5-year survival rate for late stage (III & IV) vaginal cancer is 57% and advanced disease is associated with an elevated risk of mortality (HR 1.71) [1, 10]. Due to the rare presentation, knowledge of this entity will hopefully result in early recognition so a treatment plan can be made involving radical resection with or without prior neoadjuvant therapy.
